# Three-Dimensional Vascularized Lung Cancer-on-a-Chip with Lung Extracellular Matrix Hydrogels for In Vitro Screening

**DOI:** 10.3390/cancers13163930

**Published:** 2021-08-05

**Authors:** Sangun Park, Tae Hee Kim, Soo Hyun Kim, Seungkwon You, Youngmee Jung

**Affiliations:** 1 Center for Biomaterials, Biomedical Research Institute, Korea Institute of Science and Technology (KIST), Seoul 02792, Korea; 120025@kist.re.kr (S.P.); 213872@kist.re.kr (T.H.K.); soohkim@kist.re.kr (S.H.K.); 2 Laboratory of Cell Function Regulation, Department of Biotechnology, College of Life Sciences and Biotechnology, Korea University, Seoul 02841, Korea; 3 NBIT, KU-KIST Graduate School of Converging Science and Technology, Korea University, Seoul 02841, Korea; 4 School of Electrical and Electronic Engineering, YU-KIST Institute, Yonsei University, Seoul 03722, Korea

**Keywords:** cancer-on-a-chip, tumor microenvironment, decellularized extracellular matrix, angiogenesis, vascularization, drug screening

## Abstract

**Simple Summary:**

The development of three-dimensional in vitro screening tools that mimic the microenvironment of human organs is crucial to understand the mechanism and efficacy of anti-cancer drugs in the human body. In this study, we used three strategies to engineer a three-dimensional vascularized lung cancer-on-a-chip (VLCC) to mimic the in vivo cellular microenvironment. We induced sprouting angiogenesis in the VLCC to form large blood vessels similar in size to arterioles. Additionally, we used a lung decellularized extracellular matrix to mimic the mechanical and biochemical properties of native lung tissues having various proteins and cytokines. Finally, we used tumor spheroids to mimic a solid type of lung cancer. Our drug screening tests revealed that the VLCC is a more effective tool to investigate mechanisms of drug action and their efficacies than 2D platforms. The VLCC developed in this study will improve our understanding of the effects of drug screening on the tumor environment.

**Abstract:**

Recent advances in immunotherapies and molecularly targeted therapies have led to an increased interest in exploring the field of in vitro tumor mimetic platforms. An increasing need to understand the mechanisms of anti-cancer therapies has led to the development of natural tumor tissue-like in vitro platforms capable of simulating the tumor microenvironment. The incorporation of vascular structures into the in vitro platforms could be a crucial factor for functional investigation of most anti-cancer therapies, including immunotherapies, which are closely related to the circulatory system. Decellularized lung extracellular matrix (ldECM), comprised of ECM components and pro-angiogenic factors, can initiate vascularization and is ideal for mimicking the natural microenvironment. In this study, we used a ldECM-based hydrogel to develop a 3D vascularized lung cancer-on-a-chip (VLCC). We specifically encapsulated tri-cellular spheroids made from A549 cells, HUVECs, and human lung fibroblasts, for simulating solid type lung cancer. Additionally, two channels were incorporated in the hydrogel construct to mimic perfusable vessel structures that resemble arterioles or venules. Our study highlights how a more effective dose-dependent action of the anti-cancer drug Doxorubicin was observed using a VLCC over 2D screening. This observation confirmed the potential of the VLCC as a 3D in vitro drug screening tool.

## 1. Introduction

Multiple limitations of animal studies (i.e., ethical and regulatory issues and significant differences between animal and human systems) lead us to utilize various kinds of lab-on-a-chips. To date, tissue-, cell-, and cancer-on-a-chips have been investigated to understand the biological and molecular mechanisms or screen the toxicity and efficacy of various drugs and therapies [1]. The cancer-on-chips are exceptionally high in demand, allowing us to investigate the efficacy of a diverse range of anti-cancer therapies like immunotherapies and molecularly targeted therapies with significantly variable outcomes between people [2]. There are multiple drawbacks of the conventional in vitro cancer models that try to accurately mimic the in vivo system [3,4]. Most malignant tumors undergo metastasis by releasing large quantities of cancer cells into the blood vessels that spread to distant areas [5]. In this study, we used a vascular network structure that maintains fluid flow similar to that of blood vessels or lymphatic vessels to simulate the characteristics of these cancers. The vascular networks significantly regulate normal physiological processes like metabolic activity, development, healing, immune response, and diseased physiological conditions like the progression of cancer metastases [6,7]. Therefore, incorporating vascular network structures into a cancer-on-a-chip is critical to forming a highly precise biomimicking system.

Two major processes promote the formation of vascular networks. Vasculogenesis, the *de novo* production of endothelial cells, occurs during the early developmental stages of an organism to create new blood vessels [8]. The other process similar to vasculogenesis is called angiogenesis. This multicellular morphogenetic process is regulated by a different set of genes, leading to the formation of new blood vessels sprouting from pre-existing ones [9]. Angiogenesis builds capillaries from arteries and veins, thereby establishing the circulatory connections initiated by vasculogenesis [10]. To date, multiple studies have tried to mimic the role of vascular network structures using two-dimensional (2D) substrates, 3D gels, and microfluidic devices [7,11,12,13]. Despite the partial success of such studies, they are often unstable, immature, and lack large-sized vessels for arterial and venous structures, critical for administering intravenous injections or forming solid tumor angiogenesis.

The extracellular matrix (ECM) is a complex mixture of extracellular molecules that provide biochemical and structural support to cells [14]. Decellularization involves the removal of cells, keeping intact the other ECM components in tissues or organs [15]. Decellularized ECM (dECM) produced by this technique has been used in tissue engineering applications because it mimics the natural tissue microenvironment by providing bioactive components, such as growth factors and cytokines [16,17,18,19,20]. dECM has also been used in various cancer studies as a scaffold to provide a microenvironment for the 3D culture of cancer cells. Such studies are limited to confirming only the tumor growth and proliferation due to the absence of vascular structures [21,22,23].

**Figure 1:**
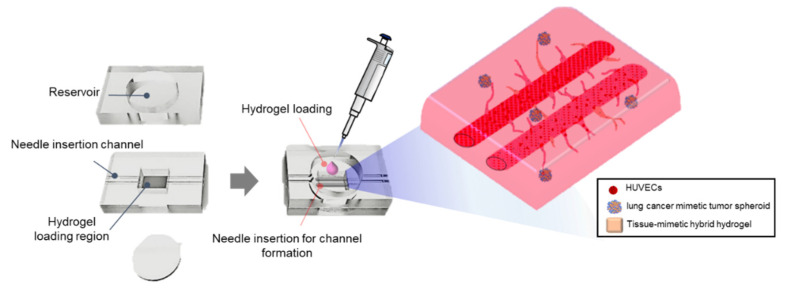
Schematic illustration of the VLCC. Lung cancer-derived tumor spheroids were encapsulated in the tissue-mimetic hybrid hydrogel to simulate the lung cancer tissue microenvironment. Two cylindrical macrochannels of 500 μM diameter were endothelialized with HUVECs inside the matrix to mimic a perfusable large-sized vessel structure.

In this study, we developed a 3D vascularized lung cancer-on-a-chip, VLCC, with the following three characteristics: (1) It is a ldECM-based pro-angiogenic tissue-mimetic hybrid (TMH) hydrogel mimicking the natural lung microenvironment, (2) It incorporates tumor spheroids composed of A549 cells, HUVECs, HLFs, to mimic solid lung cancers [13], and (3) It includes perfusable large-size vessel-like structures to simulate the functions of arteries, veins, and capillary networks [24] (Figure 1). The use of such VLCCs to evaluate drug efficacy and mechanism analysis confirmed a significant advantage over 2D screenings and 3D platforms that lack blood vessels. These results demonstrate how VLCC can potentially be a crucial 3D in vitro drug screening tool.

## 2. Materials and Methods

### 2.1. Materials and Cell Culture

PDMS (polydimethylsiloxane) was prepared using a Sylgard 184 kit (Dow Corning, MI, USA), which is a two-part PDMS system requiring a base and a curing agent mixed at a 10:1 wt. ratio [25]. RFP-HUVECs (red fluorescent protein-expressing human umbilical vein endothelial cells, passage 3) and GFP-A549 cells (green fluorescent protein-expressing adenocarcinomic human alveolar basal epithelial cells) were purchased from Angio-Proteomie (Boston, MA, USA), HUVECs (passage 3), and human lung fibroblasts (HLFs; passage 6) were from Lonza (Switzerland), and A549 cells were from ATCC (Manassas, VA, USA). RFP-HUVECs and HUVECs were grown in EBM-2 (Lonza) and EC growth supplements (SingleQuots™ Kit; Lonza, Basel, Switzerland). HLFs were grown in MEM without L-glutamine (HyClone™, UT, USA) supplemented with 10% *v/v* FBS (HyClone™), 1% *v/v* glutaMAX™-1 (Gibco, UK), 1% *v/v* sodium pyruvate (Gibco), 1% *v/v* MEM NEAA (Gibco), 0.1% *v/v* 2-mercaptoethanol (Gibco), 100 units/mL penicillin, and 0.1 mg/mL streptomycin (Gibco). GFP-A549 cells and A549 cells were grown in Roswell Park Memorial Institute medium 1640 (RPMI 1640; HyClone™) supplemented with 10% *v/v* FBS (HyClone™), 100 units/mL penicillin, and 0.1 mg/mL streptomycin (Gibco). All of the cells were cultured in a humidified atmosphere of 5% CO2 at 37 °C, and the culture media were refreshed every 2–3 days.

### 2.2. VLCC Design and Fabrication

The VLCC comprises the top layer for the reservoir, the middle layer for the in vitro 3D vascularized lung cancer tissue, and the bottom coverslip, all covalently bonded by air plasma treatment (Femto Science, Seoul, Korea). The middle and top layers of the VLCC were prepared by casting the PDMS prepolymer on the master mold fabricated using a photoresist (SU8–100, MicroChem, MA, USA). The master molds for the top and middle layers were rectangular with dimensions of 20 mm × 25 mm × 2.5 mm (width × length × height) each, while two macrochannels with a diameter of 500 µm were incorporated in the master mold for the middle layer. After the cured PDMS was peeled off from the master mold, the top layer, a reservoir for culture media, was punched out using a 15 mm diameter biopsy punch, and a chamber of size 7 mm × 5 mm × 2.5 mm (width × length × height) was cut out for the in vitro 3D vascularized lung cancer tissue. The VLCC was sterilized by autoclaving before each experiment.

### 2.3. Decellularization of Porcine Lung Tissue

Porcine lung was collected from a nearby slaughterhouse and used with approval from the supplier. For decellularization of the porcine lung tissue, chopped lung tissue was stirred in 0.1% SDS (Invitrogen, Carlsbad, CA, USA) for 120 min, followed by stirring for 10 min with 1% Triton X-100 (Biosesang, Gyeonggi-do, Korea). Antibiotic-containing PBS was used to wash the lung tissue for 3 d.

### 2.4. Biochemical Characterization of ldECM

To confirm the results of the decellularization process, histological sections were analyzed following hematoxylin and eosin (H&E), Masson’s trichrome (MT), and immunofluorescent staining. The residual deoxyribonucleic acid (DNA) and ECM components, such as collagen and glycosaminoglycans (GAGs), in the decellularized tissue were also assessed. For histological evaluation, both native and decellularized tissues were fixed in 4% paraformaldehyde (3M, Maplewood, MN, USA), embedded in paraffin, and sectioned at a thickness of 6 µm by using a microtome (RM2255; Leica, Wetzlar, Germany). Sections were deparaffinized and stained with MT to stain the collagen and sulfated GAGs as well as with H&E to stain the nucleus and cytoplasm. For the immunofluorescent staining, antibodies against COL-1 (at 1:1000; Abcam, Cambridge, UK), collagen IV (COL-4 at 1:500; Abcam), laminin (at 1:200; Abcam), and fibronectin (at 1:400; Acris, Herford, Germany) were used to identify ECM components present after decellularization.

For DNA quantification, DNA was extracted from normal and decellularized heart tissue by using a commercially available kit (DNeasy Blood & Tissue Kit; Qiagen, Venlo, The Netherlands). Extracted DNA was quantified at 260 nm wavelength using a nanodrop (ND-1000; Daemyung Science, Seoul, Korea).

The GAG content was estimated via quantifying the amount of sulfated GAGs by using 1,9-dimethylmethylene blue solution. The absorbance was measured with a microplate reader at a wavelength of 530 nm. A standard curve was created in advance by using chondroitin sulfate and was used for estimating the amount of sulfated GAGs in samples. The amount of soluble collagen was measured by using a Sircol assay kit (Biocolor, Carrickfergus, UK). Soluble collagen was extracted by applying an acid-pepsin extraction procedure as per the instruction manual provided with the assay kit and precipitated out by adding the acid-neutralizing reagent followed by isolation and addition of the concentration reagent. The precipitate was incubated with collagen dye reagent; the complex was further recovered by the addition of an alkali reagent and was measured at 555 nm. All reagents used were provided with the assay kit. The total collagen content was determined via a conventional hydroxyproline assay. The absorbance of the sample was measured at 550 nm and quantified by referring to the standard curve created in advance with hydroxyproline [26].

Furthermore, to confirm the presence of residual angiogenic cytokines in ldECM tissues, analysis of angiogenic cytokines was performed by dot-blotting using a mouse angiogenesis array kit (R&D Systems, Minneapolis, MN, USA).

### 2.5. Generation of ldECM Hydrogel and Optimization of TMH Hydrogel

Various concentrations of ldECM hydrogels were prepared following a previously published protocol but with slight modifications [20]. Lyophilized dECM was crushed into a powder using a freezer mill (6775 Freezer/Mill; SPEX SamplePrep, Metuchen, NJ, USA). ldECM powders (1, 2, and 3 *w/v*%) were solubilized using 0.5 M acetic acid with pepsin (1% *w/w* for ldECM) (Sigma-Aldrich, St. Louis, MO, USA) for 48 h. After complete solubilization of ldECM, 10X Phosphate-Buffered Saline (PBS) was added at a 1:10 ratio to the ldECM solution, and the pH was increased to 7.0, using 5M sodium hydroxide (Sigma-Aldrich). Tissue-mimetic hybrid hydrogels were prepared by mixing 1% ldECM hydrogel with 3 mg/mL of a high concentration of rat tail collagen type I solution (9.41 mg/mL; Corning, NY, USA). 1% ldECM and collagen solutions were varied in volume ratios of 25/75, 50/50, and 75/20 to optimize the tissue-mimetic hybrid hydrogel composition. Moreover, fibrinogen (3 mg/mL; Sigma-Aldrich) solution in PBS (Gibco) and the type I collagen solution were thoroughly mixed at a 1:1 ratio, and the collagen/fibrinogen solution was mixed with thrombin (50 U/mL, Sigma-Aldrich) at a volume ratio of 50:1 to use the collagen/fibrin hydrogel as a control group.

### 2.6. Evaluation of Compressive Modulus

Matrices (16 mm × 5 mm, diameter × height) for compressive modulus were prepared by loading 2 mL of 1, 2, 3% ldECM hydrogel or 25:75, 50:50, 75:25 1% ldECM/collagen hydrogel in 24 well plates and gelation at 37 °C for 30 min, respectively. The compressive modulus of the matrices was measured by performing UTM with an Instron 5966 tester (Instron, Norwood, MA, USA) at a 10N load cell number and a cross-head speed of 10 mm/min (*n* = 4). Compressive E-modulus was obtained from the stress–strain curves.

### 2.7. Scanning Electron Microscopy and Quantification of Fibers Diameter

Sample preparation for SEM was performed as follows: hydrogel matrices were fixed with 4% paraformaldehyde (3M), followed by dehydrated in 70%, 80%, 90%, 95%, and 100% ethanol solutions sequentially. The dehydrated matrices were further dried using a freezing dryer (ilshinBioBase, Gyeonggi-do, Korea) overnight. The samples were sputter-coated with gold (SPI-module sputter coater; SPI supplies, West Chester, PA, USA) and observed with a scanning electron microscope (Nova NanoSEM; FEI, Hillsboro, OR, USA) at an acceleration voltage of 15 kV. The mean fiber diameter was estimated by using Image J software and was calculated after selecting 20 single fibers randomly observed on SEM images.

### 2.8. Evaluation of Cell Viability in TMH Hydrogel

HUVECs of 10^6^ cells/mL that detached from the culture plate were centrifuged and remained cells pellet. The ldECM hydrogels or 1% ldECM/collagen hydrogels of the indicated concentration and composition ratios were added to the cells pellet, respectively. Thereafter, the suspension was pipetted into a 96 well plate and allowed to gel at 37 °C for 30 min, and then the medium was treated with each matrix and cultured for 1 and 4 days. Cell viability was assessed using a vital staining kit (Live/Dead^®^, Molecular Probes, Eugene, OR, USA). Matrices were washed three times in sterile PBS for 10 min and then incubated at 37 °C for 20 min in a solution containing 5 µL calcein-AM and 20 µL ethidium homodimer-1 in 10 mL PBS. After three subsequent PBS washes, matrices were imaged using a laser scanning confocal microscope (Carl Zeiss, Jena, Germany). Viability was quantified using Image J Software.

### 2.9. Characterization of Angiogenic Activity

The ldECM hydrogels or 1% ldECM/collagen hydrogels of the indicated concentration and composition ratios were added to the RFP-HUVECs of 10^6^ cells/mL cells pellet, respectively. Thereafter, the suspension was loaded into a 96 well plate and allowed to gel at 37 °C for 30 min, and then the medium was treated with each matrix and cultured for 7 days. The RFP-HUVECs in each matrix were imaged using a laser scanning confocal microscope (Carl Zeiss) on days 1, 4, and 7, respectively. For quantifying angiogenesis, the RFP-HUVEC images at day 7 in five random fields (200× magnification) were evaluated. The length of vascular constituent elements of angiogenesis were quantified by using Image J software (*n* = 5 in each group) [27,28].

### 2.10. Optimization of Tumor Spheroid Formation

A549 cells added to HLFs or HUVECs were used to form bi-cellular spheroids, and all three were used for forming tri-cellular spheroids to optimize the tumor spheroid formation. GFP-A549 cells, RFP-HUVECs, and HLFs, tagged with CellTracker™ Blue CMAC Dye (Thermo Fisher Scientific, Waltham, MA, USA) were used to identify each cell. The wells were first coated with a 5% Pluronic F-127 (Sigma-Aldrich) solution to prevent the cells from adhering to the plate surface. The coating was then washed off with PBS, the medium was added to the wells, and the cells were seeded. For bi-cellular spheroids, two types of cells were mixed in a ratio of 1:1, while for tri-cellular spheroids, GFP-A549 cells, RFP-HUVECs, and HLFs were mixed in a ratio of 2:1:1. The cells were then seeded in the concave microwells at a density of 1 × 10^4^ cells/well. Co-cultured and tri-cultured cells were seeded in the microwells and cultured at 37 °C with 5% CO_2_ for 3 days. The shape of the spheroids formed was observed for each condition.

### 2.11. Characterization of the VLCC

First, the tissue mimetic hybrid hydrogel was encapsulated with HUVECs (10^6^ cells/mL) and five tumor spheroids per chip. These cellular components were loaded into the chamber of the middle layer, in which channels were created using a mandrill. A thin PDMS membrane (10 mm × 10 mm × 1 mm, width × length × height) was then placed on the hydrogel-loaded chamber to prevent the hydrogel from sinking or collapsing the channel. The ldECM/collagen hydrogel was allowed to gel for 30 min at 37 °C, and then the medium was placed in the reservoir of the top layer and incubated overnight at 37 °C. After gelation, the channels formed by the mandrills were endothelialized with RFP-HUVECs at 7 × 10^6^ cells/mL, followed by rotation at 10 rotations/h for 1 h at 37 °C. The cocktail of EBM-2 complete medium, MEM complete medium, and RPMI 1640 complete medium (for HUVECs, A549, and HLFs respectively), mixed in a ratio of 2:1:1 was then placed in the top reservoir and cultured for 5 days. Images of the entire in vitro lung cancer tissue of the VLCC were acquired using tile scanning and Z-projections of the 3D stacks of a laser scanning confocal microscope (Carl Zeiss). Blood vessel lengths (*n* = 3) were measured using Image J software (National Institutes of Mental Health, Bethesda, USA).

### 2.12. Drug Administration for Drug Screening in 2D Culture, Sph-H, and VLCC

Doxorubicin (Dox; Sigma-Aldrich), an anti-cancer drug, was used for drug screening in the 2D monolayer cells (2D A549 group), the spheroids on a non-vascularized hydrogel (Sph-H group), and the VLCC. In the case of the 2D culture, cells were plated in a 24 well plate at an initial density of 5000 cells/cm^2^. For Sph-H, 300 μL of hydrogel was used in 48 wells, and 7–8 spheroids were used in proportion to the capacity. After five days of culture, treatment of 1 μM, and 5 μM Dox dissolved culture medium was given to the 2D A549, Sph-H, and VLCC groups, respectively. The 2D A549 and Sph-H groups were directly treated with Dox on the surface, whereas in the VLCC group, Dox was added to the two channels that mimicked large blood vessels. After 24 h of treatment with Dox, the protein expression patterns of the cells and the spheroids were analyzed by ELISA or fixed with 4% PFA for immunofluorescence staining.

### 2.13. The Assessment of Drug Efficacy in 2D Culture, Sph-H, and VLCC

To estimate the mechanism of DOX in VLCC, the protein level of p53, which enhances apoptosis induced by DOX, was quantified by SimpleStep ELISA^®^ Kit (ab171571; Abcam). For evaluation of apoptosis, the Cell Death Detection ELISA kit (11544675001; Sigma-Aldrich) was used. Assays were performed according to the manufacturer’s instruction [29,30,31].

Also, for immunofluorescence staining, cells or spheroids were fixed using 4% paraformaldehyde (Biosesang) in PBS for 30 min, permeabilized with 0.3% Triton X-100 (Biosesang) in PBS for 5 min, and then blocked in 4% bovine serum albumin (BSA, Sigma-Aldrich) for 2 h at room temperature. Cancer cells, endothelial cells, and apoptotic cells were marked by proSP-C (ab90716, Abcam), vWF, and caspase 3 with Alexa Fluor 594 (sc-56052, Santa Cruze Biotechnology, Inc., Santa Cruz, CA, USA) in 1% BSA respectively. Antifade Mounting Media (Vector Laboratories, Burlingame, CA, USA) was used for DAPI staining. cells and spheroids were imaged using a laser scanning confocal microscope (Carl Zeiss). Apoptosis was quantified using Image J Software (*n* = 4 per group).

### 2.14. Statistical Analysis

Experimental data were reported as mean ± standard deviation (SD). The statistical analysis was performed by using a one-way analysis of variance (ANOVA) with Tukey’s significant difference post hoc test using SPSS software (SPSS Inc., Chicago, USA). Differences were considered statistically significant when the *p*-value was lower than 0.05 (*p* < 0.05).

## 3. Results

### 3.1. Components of ldECM Shown to Be Effective in Mimicking the Native Lung Tissue

To confirm whether decellularized ldECM is practical for mimicking native lung tissue, it is necessary to investigate the composition of ldECM. First, we investigated the efficiency of the decellularization method using histological and quantitative analyses of the DNA and the ECM components of the ldECM. The hematoxylin and eosin staining (H&E) and Masson’s trichrome staining (MT) results showed that most of the nuclei, cell debris, and cytoplasm were removed in the ldECM compared with native tissue, while a large portion of collagen, one of the main ECM components, was retained in the decellularization process (Figure S1A). Additionally, we confirmed that the pro-angiogenic ECM components comprising collagen type I, type IV, laminin, and fibronectin were also present in the ldECM (Figure S1B). Quantitative analysis of DNA and the major ECM components (GAG and collagen) in ldECM showed that over 90% of the DNA was removed from the native lung tissue (95.85 ± 1.63% DNA removed in the ldECM; *p* < 0.01) after the decellularization process (Figure S1C). Of the major ECM components, GAG and collagen was retained at 73.75% ± 4.11% (*p* < 0.01) and 76.88% ± 37.34%, respectively.

Moreover, dot blot analysis of the levels of the different pro-angiogenic factors, including growth factors and cytokines, showed the presence of various pro-angiogenic factors, such as angiopoietin 1 (Ang-1), angiopoietin 3 (Ang-3), endothelin 1 (ET-1), fibroblast growth factor 1 (FGF-1), FGF-2, matrix metallopeptidase 9 (MMP-9), stromal cell-derived factor 1 (SDF-1), and vascular endothelial growth factor B (VEGF-B) in the ldECM (Figure S1D). These results show that ECM components and various growth factors can be obtained abundantly through the decellularization process. In addition, it is expected that this will better simulate the real environment of native lung tissues and help angiogenesis.

### 3.2. The Optimized Concentration of ldECM Showed A Rigid Morphology and High Cellular Activity

We then successfully prepared the matrices by varying the ldECM concentration (1, 2, and 3 wt%) to optimize the ldECM hydrogel concentration for the VLCC. Macroscopically, the structure of the matrices appeared rigid and capable of maintaining the shape towards a higher ldECM concentration (Figure S2A). Moreover, the compressive E-modulus (Young’s modulus) in the 1% ldECM group was 1.50 times lower than that of the collagen/fibrin group, and that of the 2% and 3% ldECM groups were 1.20 and 1.36 times higher than the collagen/fibrin constructs, respectively (collagen/fibrin group. vs. 1, 2, and 3% ldECM groups, respectively, *p* < 0.01) (Figure S2B). Although cell viability (90.90 ± 1.86% in the 1% ldECM group, 97.07 ± 1.17% in the 2% ldECM group, 85.62 ± 4.19% in the 3% ldECM group) was greater than 80% in all groups at day 4, it was the highest in the 2% ldECM group (Figure S2C). Also, more vessel-like structures were observed in the 1% ldECM group than in the 2% ldECM group at day 4, while no vessel-like structures were formed in the 3% ldECM group till day 7 (Figure S2D). Our investigation of the cell viability and angiogenic activity in the different ldECM groups indicated that 1% ldECM was the ideal concentration for further use.

### 3.3. The Ratio of ldECM/Collagen Composition Having Good Performances Was Optimized

**Figure 2:**
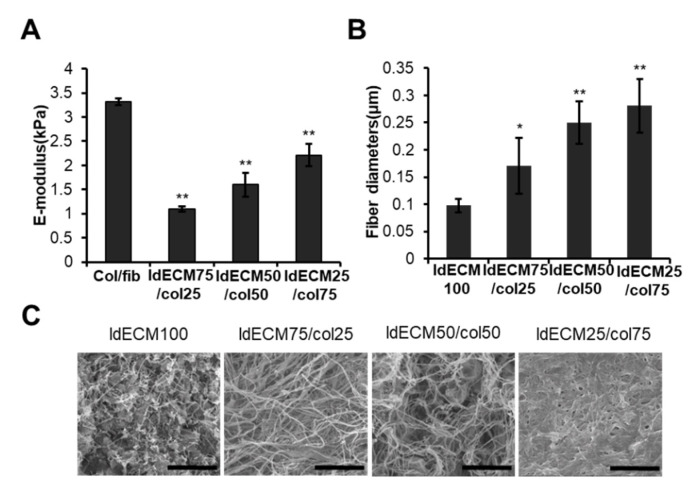
Characterization of TMH hydrogels based on the composition of the 1% ldECM/collagen matrices. (**A**) Compressive E-modulus (Young’s modulus, kPa) of col/fib and 1% ldECM/collagen matrices at various indicated composition. ** *p* < 0.01 compared with the col/fib group. (**B**) Quantification of fiber diameters in 1% ldECM and 1% ldECM/collagen matrices at various indicated compositions. * *p* < 0.05, ** *p* < 0.01 compared with the 1% ldECM 100 group. (**C**) SEM images of 1% ldECM and 1% ldECM/collagen matrices at various indicated compositions.

We prepared tissue-mimetic hybrid (TMH) hydrogel at ldECM/collagen ratios of 25:75, 50:50, and 75:25 (%) to increase the stiffness of the 1% ldECM hydrogel to maintain its structure in the VLCC. Optical imaging revealed that all the ldECM/collagen groups were soft and swollen compared with the collagen/fibrin matrix and the ldECM/collagen matrices became rigid, clear edges with a higher content of collagen (data not shown). The compressive E-moduli (3.32 ± 0.07 kPa in collagen/fibrin group, 1.09 ± 0.06 kPa in the 75:25 ldECM/collagen group, 1.60 ± 0.24 kPa in the 50:50 ldECM/collagen group and 2.17 ± 0.27 kPa in the 25:75 ldECM/collagen group) were significantly lower in all ldECM/collagen groups than in the collagen/fibrin group (*p* < 0.01). The 25:75 ldECM/collagen group had the highest E-modulus among the ldECM/collagen groups (Figure 2A).

SEM images of 1% ldECM and TMH hydrogel revealed that the 1% ldECM matrix had a microstructure with many masses embedded in networks of fine fibers, while TMH hydrogel had few masses and consisted more of a fibrous network (Figure 2C). Interestingly, the thickness of the fibers constituting the microstructure of TMH hydrogel increased with an increase in the collagen ratio. Moreover, quantitative analysis showed that the fiber diameters of ldECM/collagen compositions of 75:25, 50:50, and 25:75 groups were 1.75, 2.56, and 2.88 times greater than pure ldECM matrix, respectively (Figure 2B; 1% ldECM-100 group vs. 75:25 ldECM/collagen group, *p* < 0.05; 1% ldECM-100 group vs. 50:50 and 25:75 ldECM/collagen groups, *p* < 0.01). This result shows that the collagen fiber increases by the collagen concentration, and thus the structure can be maintained more stably.

**Figure 3:**
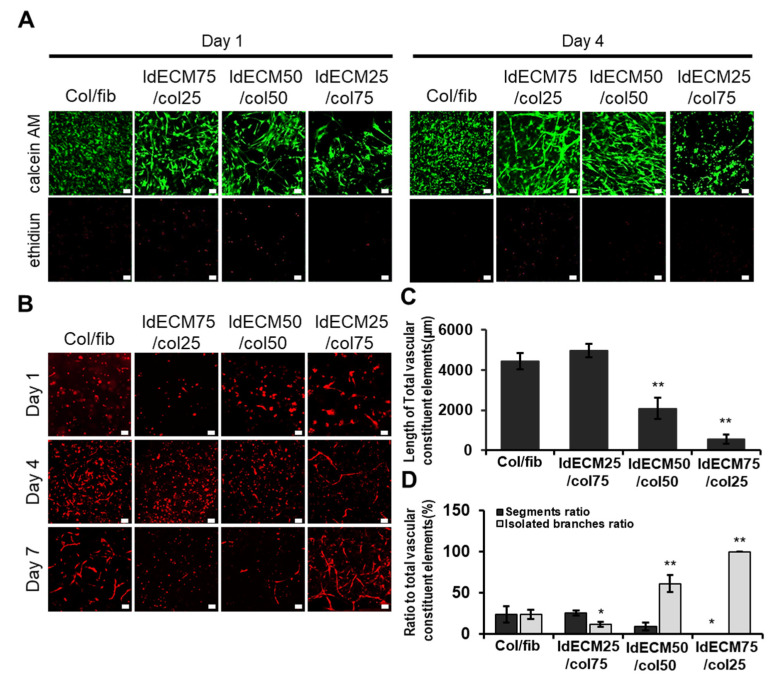
Analysis of cell viability and angiogenic potential in TMH hydrogels based on the composition of the 1% ldECM/collagen matrices. (**A**) Representative images indicating cell viability on days 1 and 4, and (**B**) vessel-like structures on days 1, 4, and 7 in 1% ldECM/collagen matrices as indicated. Scale bars: 50 µm (A), 100 μM (B). (**C**) Quantification of total vascular constituent elements of angiogenesis. ** *p* < 0.01 compared with the col/fib group. (**D**) Quantification of segments and isolated branches to total vascular constituent elements. * *p* < 0.05, ** *p* < 0.01 compared with col/fib group.

We observed that the cell viability was greater than 90% in the 25:75 and 50:50 ldECM/collagen groups, while that of the 25:75 ldECM/collagen group was 99.12%, which was similar to the collagen/fibrin group (98.02 ± 0.81%). We also found that the 75:25 ldECM/collagen group had the lowest cell viability (approximately 70%) (Figure 3A). The sprouting of HUVECs in the 25:75 ldECM/collagen group began from day 1 to form a complex capillary network-like structure similar to that of the collagen/fibrin group on day 7, whereas the sprouting of the HUVECs was the least in the 75/25 group, after 7 days (Figure 3B). We investigated constituent elements of angiogenesis on day 7. The constituent elements can be classified into segments, branches, and isolated branches, respectively, representing mature blood vessels, intermediate blood vessels, and immature blood vessels. As shown in Figure 3C, Col/fib and 25:75 ldECM/collagen groups showed similar degrees, and the other two groups showed low total length. As a result of checking the ratio of the segment and isolated branches, high levels of segment ratios were obtained in the Col/fib and 25:75 ldECM/collagen groups. In the other two groups, it was confirmed that the ratio of segment elements was low while the ratio of isolated branches was high (Figure 3D). These observations indicated the 25:75 ldECM/collagen composition to be the most optimal for the VLCC.

### 3.4. A More Compact and Angiogenic Spheroid Was Produced through Tri-Cellular Culture

**Figure 4:**
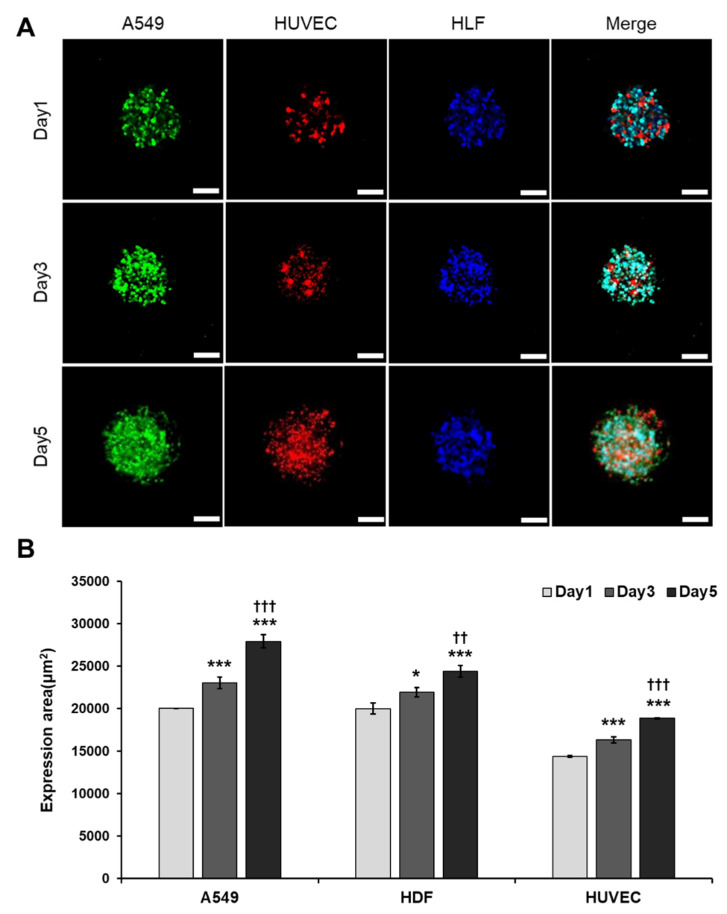
Optimization of tumor spheroid formation. (**A**) Fluorescent image of tri-cellular spheroids at 1, 3 and 5 days after seeding. Scale bar: 100 μM. (**B**) Quantification of expression area of A549 cells, HUVECs and HLFs. * *p* < 0.05, *** *p* < 0.005 compared with Day 1, †† *p* < 0.01, ††† *p* < 0.005 compared with Day 3.

Through previous studies, it was confirmed that multicellular culture could better simulate the cancer microenvironment and induce angiogenesis more easily. We used GFP-A549 cells, RFP-HUVECs, and CMAC-stained HLFs to mimic solid tumors like lung cancer [13,32]. Concave microwells were used to form size-controlled spheroids [33] which make more constant results. To induce blood vessels extending from the inside of the spheroid, GFP-A549 cells and RFP-HUVECs were co-cultured in a 1:1 ratio. We observed that HUVECs co-culture led to cell dispersion and failed to form spheroids (Figure S4A). On the other hand, the co-culture of HLFs with the same ratio showed the formation of a compact circular spheroid just one day after seeding (data not shown). We decided to use a multicellular culture method using RFP-HUVECs that induce angiogenesis from the inside and HLFs that induce cell aggregation and produce growth factors. The primary criteria to be eligible to form the device include the ability to induce angiogenesis and for the formation of solid tumors that are not easily degraded. To maintain the characteristics of lung cancer spheroid, the ratio of GFP-A549 cells was set to 50% and the ratio of the remaining cells was mixed equally. Keeping this fact in mind, we decided to test our tri-culture model with GFP-A549 cells, RFP-HUVECs, and HLFs at a 2:1:1 ratio. Our tri-cellular conditions led to a spheroid formation with GFP-A549 cells firmly located on the outside, with a mixture of RPF-HUVECs and HLFs on the inside (Figure 4A). All three types of cells were able to proliferate and maintain their morphology by day 5 (Figure 4B). Angiogenesis from the inside of the spheroid was observed on day 5 (Figure 4A).

### 3.5. Angiogenic Sprouting Was Confirmed in VLCC under Optimized Conditions

**Figure 5:**
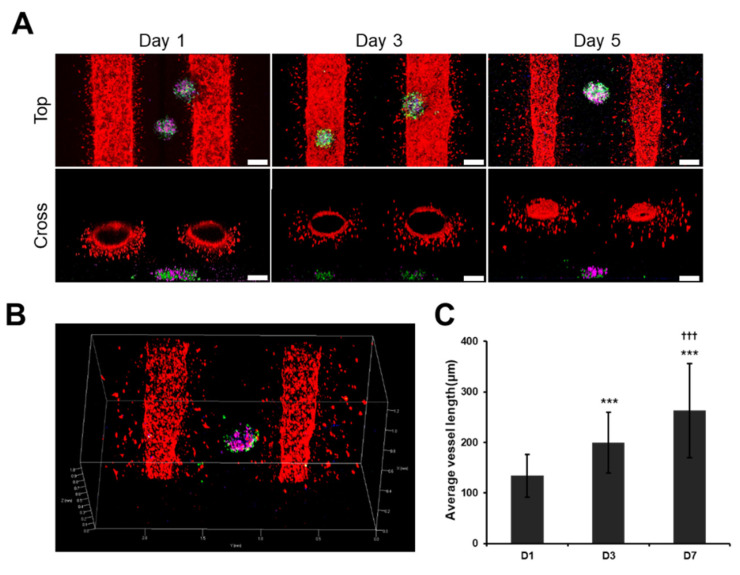
Angiogenic sprouting in VLCC. (**A**) Representative confocal microscope images from the top and cross-section of VLCC on day 1, 3, and 5. (**B**) z-stack images on day 5 (red color denotes the HUVECs in the channel, green color denotes the A549 cells in the spheroids, blue color denotes the HLFs in the spheroids, magenta color denotes the HUVECs in the spheroids). Scale bar: 200 μM. (**C**) Quantification of the average length of blood vessels. *** *p* < 0.005 compared to D1, ††† *p* < 0.005 compared to D3.

We devised the VLCC using our optimized conditions of matrix formation using 1% ldECM and a 25:75 ldECM/collagen composite hydrogel and compared with it the collagen/fibrin gel (3 mg/mL collagen, 3 mg/mL fibrin at a ratio of 1:1). We used GFP-A549 cells and RFP-HUVECs to determine the relationship between the formation of cancer spheroids and vessel expansion from the macrochannels (large-sized vessels). We observed that the shape was retained after gel loading in the TMH hydrogel but shrank in the case of collagen/fibrin hydrogels within one day of gel loading (Figure S3). Hence, we used the TMH hydrogels in the subsequent experiments, unless otherwise mentioned. Our investigations revealed that tight cell-cell interactions in the endothelialized ECs formed large-sized vessels in the two macrochannels, while angiogenic sprouting led to vasculature formation between the two vessels (Figure 5A,B). In addition, the average length of the blood vessels extending in various directions on day 1 was 134.27 ± 41.92 μM, and later increased to 199.51 ± 60.54 μM and 263.18 ± 93.15 μM on day 3 and day 7, respectively (Figure 5C). Moreover, the angiogenic vessels extended towards the direction of the spheroids from the large vessels over time. This shows that angiogenesis that occurs in a native cancer microenvironment can be simulated, suggesting the possibility of drug delivery through the connected blood vessels.

### 3.6. VLCC Responded MORE Efficiently for Drug Efficacy Evaluation and Mechanism Estimation

**Figure 6:**
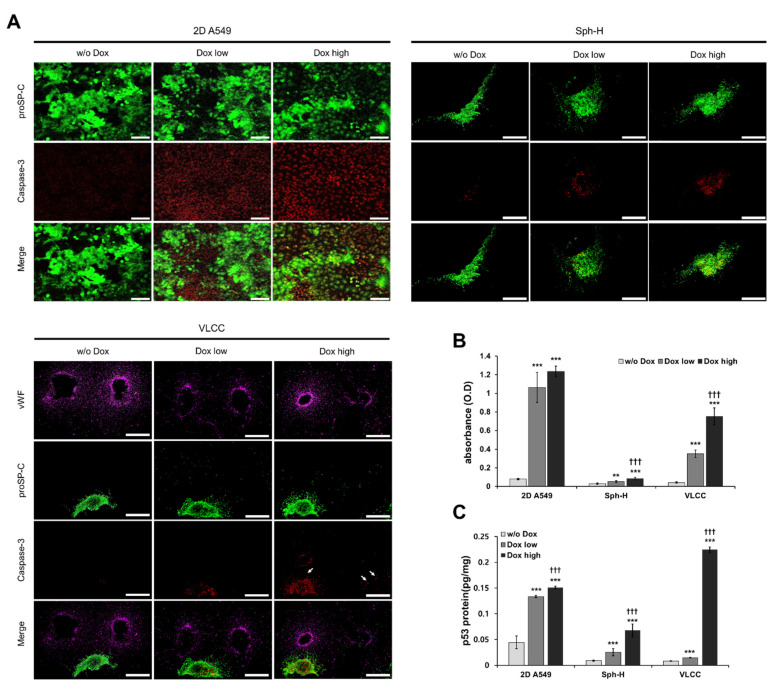
Evaluation of drug efficacy using Doxorubicin. (**A**) Representative immunofluorescence images of apoptotic cells on 2D A549, Sph-H, and the VLCC with different Dox doses. Scale bar: 100 μM (2D culture), 500 μM (Sph-H, VLCC). (**B**) Quantification of the cell death in 2D A549, Sph-H, and the VLCC with different Dox doses. ** *p* < 0.01, *** *p* < 0.005 compared with w/o Dox, ††† *p* < 0.005 compared with Dox low. (**C**) Quantification of the p53 protein level in 2D A549, Sph-H, and the VLCC with different Dox doses. *** *p* < 0.005 compared with w/o Dox, ††† *p* < 0.005 compared to Dox low.

Next, we assessed the caspase-3 activity by treating the 2D cell cultures, spheroids with non-vascularized hydrogel (Sph-H), and the VLCC with Doxorubicin (1 μM, Dox low and 5 μM, Dox high) and without Doxorubicin, for each condition to evaluate drug efficacy. In Figure 6, vWF represents HUVECs and Pro-SPC represents A549 cells, which show blood vessels and spheroids in VLCC, respectively. Dox was our drug of choice because of its effectiveness and widespread use in cancer treatment [34]. 2D A549 cells showed the highest caspase-3 activity with no significant dose-dependent differences (Figure 6A-2D A549). In Sph-H, the caspase-3 activity was lower than that in 2D A549 cells, with no significant dose-dependent differences (Figure 6A-Sph-H). On the other hand, in the VLCC, caspase-3 activity indicated reduced apoptosis while maintaining a higher level of caspase-3 activity than Sph-H, with significant dose-dependent differences (Figure 6A-VLCC). These results show that VLCC may respond more sensitively to drug concentration when compared to 2D A549 and Sph-H.

ELISA-mediated detection of cell death more clearly showed significant differences between each group (Figure 6B). In the case of 2D A549 cells, increased apoptosis was observed in both Dox high and Dox low (1.06 ± 0.16 and 1.24 ± 0.06, respectively), with insignificant differences in their values. Apoptosis was drastically reduced in the case of Sph-H, compared with that of 2D A549 cells. Finally, the apoptosis in the VLCC in both Dox low and high conditions was higher than that of Sph-H (0.35 ± 0.04 and 0.75 ± 0.09), with significant dose-dependent differences (*p* < 0.005). Furthermore, the Dox high condition in the VLCC led to an induction of apoptosis and a decrease in angiogenesis in the tumor spheroids (Figure 6A, white arrow). This suggests that it can also show the effect of drugs on angiogenesis.

Next, we focused on p53, a known inducer of DNA damage, thereby causing cell cycle arrest and apoptosis. Dox-treatment induces the p53 signaling pathway, leading to DNA damage and apoptosis [34,35]. To evaluate the mechanism of anti-cancer drugs, we confirmed whether Dox-treatment changes the level of p53 protein expression in the VLCC. We observed that increasing the Dox concentration led to increased levels of p53 in all three conditions (Figure 6C). In the 2D A549 cells, the increase in p53 level was 1.1 times, while it increased by 2.2 times and 16.1 times in Sph-H and VLCC, respectively. These results collectively prove the heightened dose-dependent expression of p53 in the VLCC compared with the 2D A549 and Sph-H conditions.

## 4. Discussion

Various studies have attempted to develop in vitro cancer mimetic platforms to investigate the mechanism of metastasis, which contributes majorly to high cancer-related mortality. Such mimetic platforms also aim to evaluate the efficacy of novel immunotherapies and molecularly targeted agents [3]. A popular belief is that the simulation of the natural tumor microenvironment and the formation of vascular structures are essential for developing more accurate in vitro cancer mimetic platforms [36]. In this study, we focused on the development of VLCC that most closely simulates native lung cancer tissue by combining ldECM-based tissue-mimetic hybrid hydrogels and lung cancer-mimetic tumor spheroids. It also incorporates large vascular and capillary networks to mimic the cancer system closely. Our results showed that ldECM comprises pro-angiogenic factors and various necessary ECM components of the natural lung tissue. It is known that MMP-9 particularly enhances angiogenesis via an angiogenic switch, while Ang-1 and VEGF-B play crucial roles in the maintenance and maturation of newly formed vessels [37,38,39]. Thus, we chose ldECM as a significant component of a composite hydrogel used in our chip to simulate the vascularized natural lung cancer tissue.

Next, we investigated the mechanical and biological properties of 1, 2, and 3 wt% of the ldECM compared with the collagen/fibrin hydrogels to optimize the concentration of the ldECM hydrogel for the VLCC. Collagen and fibrin hydrogels, the major constituents of the ECM, are commonly used for biomimetic microenvironments in angiogenesis and cancer research. Hence, we used a collagen/fibrin hydrogel as a positive control in this study [24,40]. The compressive modulus or the stiffness of the material increased with an increasing concentration of ldECM, with 2 and 3 wt% concentrations being stiffer than the collagen/fibrin hydrogel. Our study revealed that the concentration-dependent pro-angiogenic potential of ldECM increased even at lower concentrations, with 1% ldECM showing significant enhancement compared with the collagen/fibrin hydrogel.

On the other hand, we observed that the cell viability was highest at 2% and lowest at 3% concentration. Concentration-dependent stiffness and pro-angiogenic potential of ldECM showed a negative correlation with cell viability. These results corroborate the findings from recent studies highlighting how angiogenic properties are inhibited by stiffer materials [24,41,42]. Cell viability was highest at 2% ldECM, which showed the most similar stiffness to the collagen/fibrin hydrogel. These results collectively suggest how material stiffness can modulate cell viability.

We found that 1 wt% concentration of ldECM was optimal for our study with the highest pro-angiogenic activity. However, low cell viability and stiffness remain to be limitations of the VLCC. Incorporating large-sized vessel-like structures into VLCC was particularly challenging with hydrogels of low stiffness (data not shown). Thus, we developed TMH hydrogels in which the collagen hydrogel and ldECM were combined, and the optimal conditions were explored to complement the cell viability and stiffness. We observed that the TMH hydrogels composition, stiffness, cell viability, and angiogenesis were highest when ldECM and collagen were mixed in a ratio of 25:75. Although cell viability and the pro-angiogenic activity were higher in the 25:75 ldECM/collagen group than in the 1% ldECM-100 group, the stiffness was not significantly different (E-modulus of 1% ldECM-100 = 2.21 ± 0.23 kPa, 25:75 ldECM/collagen = 2.17 ± 0.27 kPa). However, we hypothesized that the difference in microstructure between the two hydrogel groups possibly affected their cell viability and the pro-angiogenic activities. The 1% ldECM-100 group microstructure showed numerous masses embedded in fine fiber networks, while the 25:75 ldECM/collagen group lacked such masses but formed a dense and thick fibrous network. Some recent studies propose how the fiber diameter directly affects fiber stiffness. However, the volume fraction rather than the fiber thickness dominates the bulk mechanical properties [43,44].

In this study, we constructed the 3D VLCC via optimized mechanical/biological properties of the ldECM-based TMH hydrogels and successfully established the lung cancer spheroid formation protocol. The VLCCs formed had large stable vascular channels with capillary network structures between channels. This model is distinct from the vessel-like structures developed by other groups [45]. Other models are flatter than full 3D models and cannot show angiogenesis sprouting from pre-existing blood vessels. However, our platform can better simulate the real environment because capillaries extend from blood vessels of a complete 3D structure. Our investigations showed angiogenic sprouting in several directions on day 1 in response to the numerous pro-angiogenic growth factors and cytokines present in the VLCCs composed of ldECM [18,46]. Additionally, it is also known that the vessel maturation potential of angiopoietin in ldECM positively affects vessel maturation and long-term blood vessel formation [47,48]. As shown in Figure 5, the lengths of the new blood vessels directed towards the spheroids increase with time. This occurrence is because new blood vessels initially formed due to the effect of pro-angiogenic growth factors and cytokines, which respond to new growth factors secreted by the spheroids. Therefore, a gradient created by the growth factors secreted by the spheroids affects the direction of blood vessel formation [49,50]. These results show the possibility of substance transport and drug delivery into the blood vessels. If drug screening is possible through blood vessels connected to spheroids, more specific results will be obtained.

Next, we performed a drug screening using Dox, which has already been published on effects, side effects, and doses in various groups. We set the dose by referring to published studies and evaluated whether our developed VLCC responds effectively to the drug [51,52,53]. The results show that the drug is effectively delivered in the VLCC similar to native tumors with a significant dose-dependent variation in its activity. As shown in Figure 6B, the 2D A549 group showed increased apoptosis than the Sph-H and the VLCC groups upon treatment with high and low amounts of Dox, with minimal dose-dependent differences. On the other hand, the Sph-H and VLCC groups showed decreased apoptosis than in the 2D A549 group but showed increased dose-dependent differences. In the Sph-H group, the buffering action of the TMH hydrogel sharply decreased the overall apoptosis by inhibiting the direct drug effect. On the other hand, in the VLCC, the decrease in apoptosis due to the buffering action was not as drastic as in the Sph-H group but showed significant dose-dependent differences. This observation can be attributed to the specific effect of the drug on the tumor spheroids due to the formation of vascular structures leading to angiogenesis. Additionally, Dox is known to exhibit anti-angiogenic effects [54]. Moreover, as shown in Figure 6A, high Dox conditions led to the apoptosis of angiogenic endothelial cells in the VLCC group. These results collectively suggest that the VLCC is more sensitive to dose-dependent drug effects than the 2D cell culture systems and non-vascularized 3D culture systems. We also propose that the VLCC is a potential platform to evaluate the various actions of anti-cancer drugs such as the anti-angiogenic ones.

We also quantitatively evaluated the role of Dox on modulating p53 protein levels in the different groups (Figure 6C). The cytostatic drug Dox induces p53 signaling, leading to DNA damage and apoptosis [34,35]. The Dox-dependent expression changes of p53 were analyzed in each group to evaluate the potential of the VLCC in mechanistic studies. The expression of p53 similarly in each group showed a similar trend to that of the level of apoptosis. In the 2D A549 group, the expression of p53 was high regardless of the dose. In contrast, the Sph-H group showed variable (both low and high) expression levels of p53. The VLCC group showed a Dox dose-dependent expression of p53. This observation proved that the VLCC had the highest sensitivity towards Dox and that it can be efficiently used for mechanism analysis by investigating the differences in the expression of related proteins. Because our model has a vascularization model, we will be able to investigate the mechanism of anti-angiogenic agents through future studies analyzing factors such as VEGF, bFGF, and PIGF [55].

In this study, we conclusively highlight the potential of the VLCC as an in vitro platform for more accurate drug screening by mimicking the native lung cancer microenvironment. Future studies are required to incorporate microfluidics simulating blood flow into our proposed platform. Such in-depth analysis would help to extend the utility of this platform immunotherapy and metastasis studies.

## 5. Conclusions

This study developed a VLCC that closely mimics the natural lung tumor microenvironment for drug screening. We developed our VLCC system to include a large vessel-like structure and a natural lung cancer tissue-like microenvironment using lung cancer spheroids and ldECM. We confirmed that angiogenesis and cell viability were enhanced by regulating the mechanical and biochemical properties of the ldECM-based TMH hydrogel that depends on hydrogel ratio and concentrations. Through this ldECM-based TMH hydrogel, we could successfully create a natural lung cancer tissue-like structure. Additionally, the tri-cellular spheroid formation process was optimized to simulate actual solid lung cancers. The 3D VLCC developed in this study successfully showed the angiogenesis process in the tumor microenvironment. Furthermore, the effectiveness of the VLCC was demonstrated in terms of drug efficacy and mechanism analysis compared with 2D cells and 3D platforms lacking vascular structures. We believe that the VLCC system enables more accurate drug screening tests and can be used as an in vitro tool to complement animal testing in the preclinical stage.

## Data Availability

Any raw data presented in this study is available on request to the corresponding author. Supporting information is available in the attached Supplementary Files.

## Supplementary Materials

The following are available online at https://www.mdpi.com/article/10.3390/cancers13163930/s1, Figure S1: Component analysis of ldECM. Figure S2: Characterization of various ldECM concentrations on matrix formation. Figure S3: Developing the VLCC using tissue-mimetic hybrid hydrogel. Figure S4: Optimization of tumor spheroid formation.
