# Supplementary material for: Three-Dimensional Vascularized Lung Cancer-on-a-Chip with Lung Extracellular Matrix Hydrogels for In Vitro Screening
Source: Cancers (Basel). 2021 Aug 5;13(16):3930. doi: 10.3390/cancers13163930 (PMC8393390; doi:10.3390/cancers13163930)
Supplement: Supplementary file 1 [file cancers-13-03930-s001.zip › cancers-1293640-supplementary.pdf]

cancers

MDPI

Article

Supplementary File:

Three-dimensional Vascularized Lung Cancer-on-a-chip with Lung Extracellular Matrix Hydrogels for *In Vitro* Screening

Sangun Park[1,2,†], Tae Hee Kim[1,†], Soo Hyun Kim[1,3], Seungkwon You[2,*], Youngmee Jung[1,4,*]

1 Center for Biomaterials, Biomedical Research Institute, Korea Institute of Science and Technology (KIST), Seoul, Republic of Korea; 120025@kist.re.kr(S.P.); 213872@kist.re.kr(T.H.K.); soohkim@kist.re.kr(S.H.K); winnie97@kist.re.kr(Y.J.)
2 Laboratory of Cell Function Regulation, Department of Biotechnology, College of Life Sciences and Biotechnology, Korea University, Seoul 136-701, Republic of Korea;120025@kist.re.kr(S.P.); bioseung@korea.ac.kr(S.Y.)
3 NBIT, KU-KIST Graduate School of Converging Science and Technology, Korea University, Seoul, 02841, Republic of Korea; soohkim@kist.re.kr(S.H.K)
4 School of Electrical and Electronic Engineering, YU-KIST Institute, Yonsei University, Seoul, Republic of Korea; winnie97@kist.re.kr(Y.J.)
* Correspondence: bioseung@korea.ac.kr(S.Y.); winnie97@kist.re.kr; Tel.:82-2-958-5348(Y.J.)
† Equal contributions.

Supplementary Figure S1

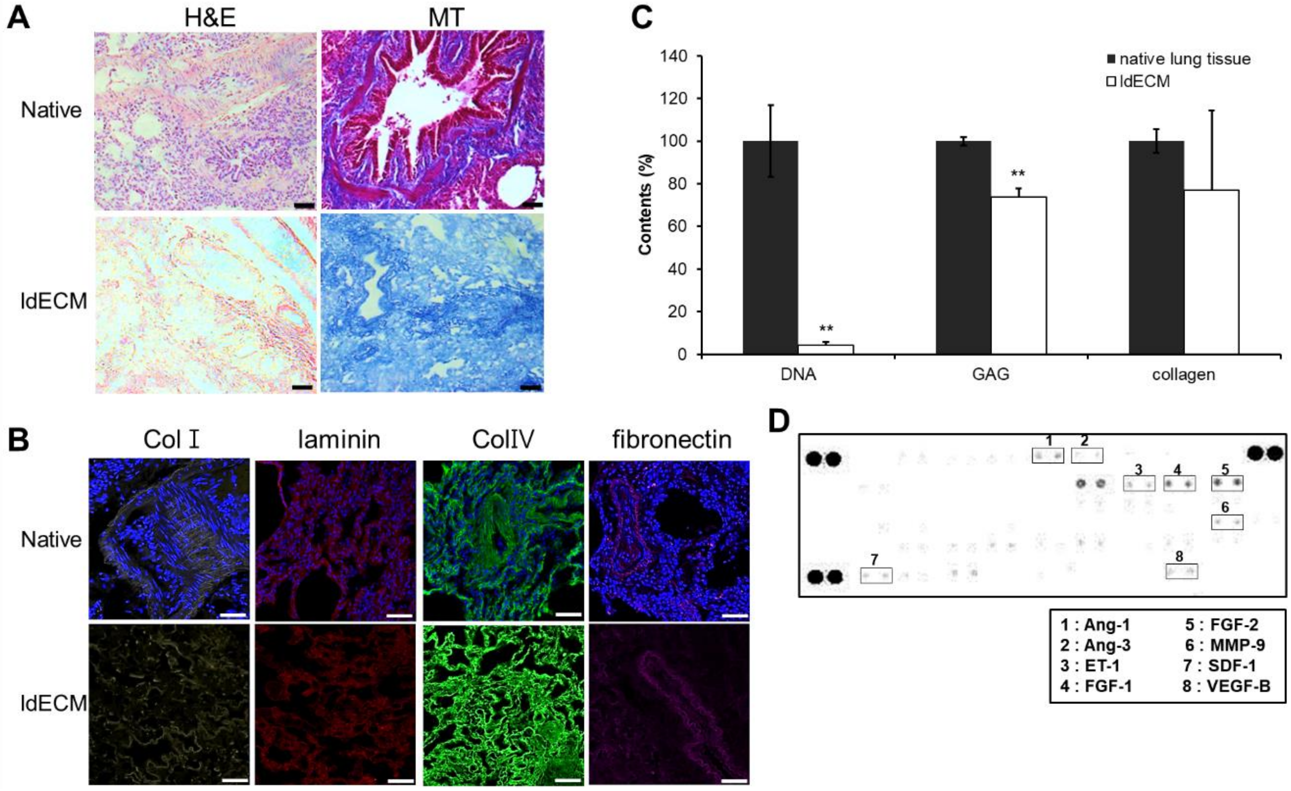

Figure S1. Component analysis of ldECM. (A) H&E and MT staining of native and ldECM. Scale bars: 100 μm. (B) Immunofluorescence staining of the ECM components Col-I and IV, laminin, and fibronectin. Scale bars: 50 μm. (C) Quantification of DNA contents and ECM components (GAGs and collagen) of native ECM and ldECM. **p < 0.01. (D) Analysis of pro-angiogenic factors present in the ldECM.

Supplementary Figure S2

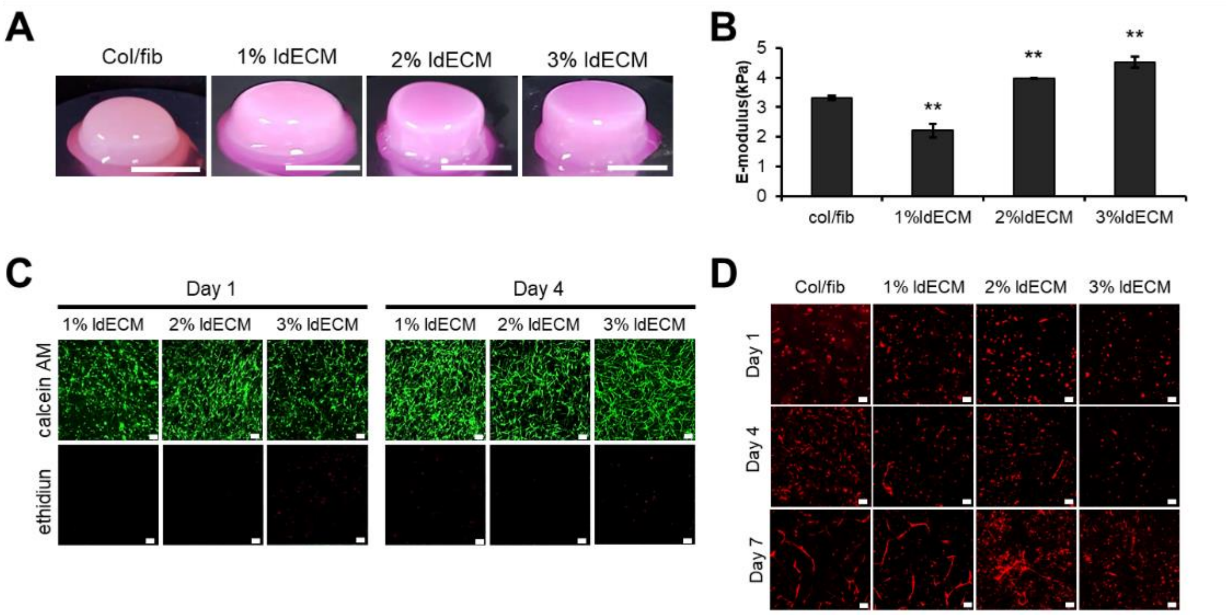

Figure S2. Characterization of various ldECM concentrations on matrix formation. (A) Images of 50/50 collagen/fibrin (col/fib) and ldECM matrices at different indicated concentrations. Scale bars: 10 mm. (B) Quantification of compressive E-modulus (Young's modulus, kPa) of ldECM matrices. **$p < 0.01$ compared to the col/fib group. (C) Representative images of cell viability assay on days 1 and 4, and (D) vessel-like structures on days 1, 4, and 7 in ldECM matrices of different indicated concentrations. Scale bars: 50 μm(C), 100μm(D).

Supplementary Figure S3

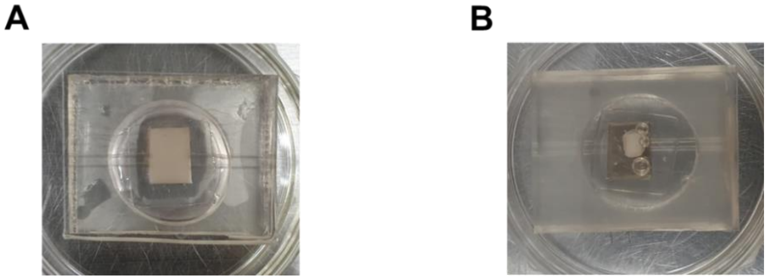

Figure S3. Developing the VLCC using tissue-mimetic hybrid hydrogel. (A) Collagen/fibrin hydrogel (3 mg/ml collagen, 3 mg/ml fibrin at ratio of 1:1). (B). Representative images of the hydrogel one day after gel loading.

Supplementary Figure S4

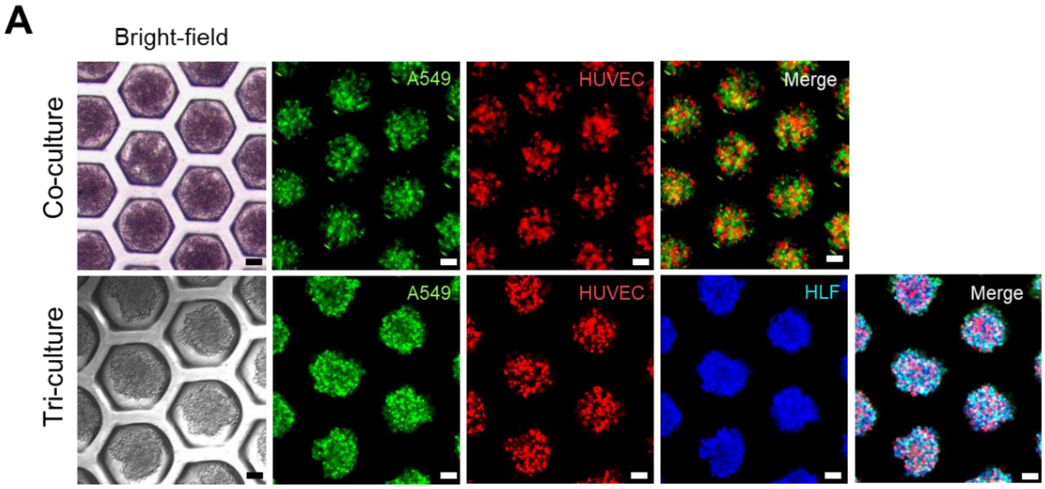

Figure S4. Optimization of tumor spheroid formation. (A) Representative bright-field and fluorescent images of bi-cellular and tri-cellular spheroids formed in the Stem fit micromolds one day after seeding. Scale bar: 100 μm.
